# Immersive Patient Education in Radiation Oncology: A Prospective, Randomized, Feasibility Pilot Study

**DOI:** 10.7759/cureus.113287

**Published:** 2026-07-24

**Authors:** Sydney Lash, Sarah B Wisnoskie, Sam Hendley, Michelle Rokni, Nicole Carone, Sarah Cummings, Christy Hickerson, Gretchen Kessler, Kelli Reardon, Alison Amos, Ashlyn Zebrowski, Alan Baydush

**Affiliations:** 1 Carolina Health Informatics Program, The University of North Carolina at Chapel Hill, Chapel Hill, USA; 2 Radiation Oncology, Novant Health Forsyth Medical Center, Winston-Salem, USA; 3 Radiation Oncology, City of Hope, Los Angeles, USA; 4 Radiation Oncology, University of North Carolina Health Care, Chapel Hill, USA

**Keywords:** external beam radiation therapy, immersive medical education, patient education (topic), patient experience, virtual reality (vr)

## Abstract

Background and objective

Patients undergoing external beam radiation therapy (EBRT) frequently experience anxiety, uncertainty, and difficulty understanding the treatment process. Immersive educational approaches incorporating 360° video technologies may improve patient understanding and preparedness while enhancing the overall patient experience. This study aimed to prospectively evaluate the operational feasibility and patient experience outcomes associated with implementation of a flexible immersive patient education program (IPEP) integrated into routine radiation oncology workflows for patients undergoing EBRT.

Methods

A prospective randomized pilot study was conducted to compare standard-of-care (SOC) education with an immersive education intervention integrated into routine radiation oncology workflows. Immersive educational materials consisting of 360° first-person-perspective videos were delivered through either a virtual reality (VR) headset or a flattened video format based on patient preference and tolerance. Patient-reported anxiety, understanding, and satisfaction were assessed longitudinally throughout treatment using Likert-scale survey instruments. Treatment workflow metrics including treatment time, imaging frequency, and setup shifts were also evaluated.

Results

Forty-nine patients completed the study (24 SOC and 25 intervention: eight VR, 17 flattened first-person-perspective video). The intervention group demonstrated greater reductions in treatment-related, cancer-related, and care plan-related anxiety throughout treatment. Reduction in care plan-related anxiety was significantly greater in the intervention arm (p = 0.01). Patient-reported understanding improved significantly in the intervention group compared with standard of care (p = 0.03). Satisfaction improved in both groups, with larger positive changes observed in the intervention arm. Exploratory workflow metrics demonstrated directional trends toward improved treatment efficiency in the intervention arm, including reductions in treatment time, imaging frequency, and number of setup shifts over the treatment course.

Conclusions

The implementation of an IPEP was associated with improvements in patient-reported anxiety and understanding during radiation therapy. These findings support the feasibility of integrating an IPEP into routine radiation oncology workflows and suggest potential benefits for patient experience outcomes. Larger studies are needed to determine the effectiveness of immersive education approaches and their impact on workflow efficiency.

## Introduction

Patients undergoing radiation therapy frequently experience anxiety, uncertainty, and difficulty understanding the treatment process. Radiation oncology workflows involve complex treatment planning, immobilization devices, advanced imaging, and highly technical treatment delivery systems that can be unfamiliar to patients. Prior studies have demonstrated that anxiety and limited understanding during treatment can negatively affect patient experience, satisfaction, and perceived preparedness for care [1-5]. 

Educational interventions have been shown to improve patient understanding and reduce anxiety in oncology settings [3,6-9]. Video-based educational approaches may be particularly valuable because they provide standardized information delivery while allowing patients and caregivers to review educational content outside of physician encounters. However, conventional two-dimensional educational approaches may be limited in their ability to effectively convey the spatial and procedural aspects of radiation therapy. 

Immersive educational technologies, including virtual reality (VR) and 360-degree visualization platforms, have increasingly been explored within healthcare education [3,5,10-14]. Prior studies in radiation oncology have demonstrated improvements in patient understanding, engagement, and satisfaction following immersive educational interventions [3,5,10-14]. Despite encouraging early findings, many previously published studies have been limited by small sample sizes, single-arm designs, or the evaluation of immersive technologies outside routine clinical workflows.

This study sought to prospectively evaluate the feasibility and patient experience outcomes associated with the implementation of an immersive patient education program (IPEP) integrated into routine radiation oncology care. We hypothesized that the addition of the IPEP to standard-of-care (SOC) education would improve patient understanding and reduce patient-reported anxiety compared with SOC education alone.

## Materials and methods

Study design****

This prospective pilot feasibility study enrolled eligible patients over a predefined six-month period. The enrollment period was determined by the project timeline and available resources rather than by a target sample size. As the primary objective was to evaluate the feasibility and preliminary impact of the IPEP, no a priori power calculation was performed. Patients were randomized to receive either SOC education or an immersive educational intervention integrated into routine clinical workflows. Patients were randomized using simple randomization based on a coin flip before enrollment. Study identification numbers were preassigned to either the SOC or intervention arm according to the randomization sequence, and enrolled patients were assigned the next available study identification number after obtaining consent.

This process ensured that treatment assignment was determined by a predetermined randomization sequence without investigator discretion at the time of enrollment. SOC education consisted primarily of physician-delivered education during the initial radiation oncology consultation. This discussion included information regarding the patient's diagnosis, recommended radiation treatment, anticipated treatment process, and potential side effects. No standardized supplemental educational materials were provided as part of SOC during the study period. As physicians did not know which arm a patient would be randomized to at the time of the consultation, all patients received the SOC education provided during the clinical consultation. Patients randomized to the intervention arm subsequently received the IPEP in addition to SOC education. Therefore, this study evaluated the incremental benefit of adding the IPEP to the existing patient education workflow.

Feasibility outcomes included successful integration into routine clinical workflow, patient completion of educational sessions, modality utilization patterns, tolerability of immersive content, and scalability of educational delivery across treatment pathways. Exploratory secondary outcomes included longitudinal patient-reported measures of anxiety, understanding, satisfaction, and treatment workflow metrics intended to support hypothesis generation for future studies. The study protocol was approved by the institutional review board (approval No. 24-2626). The project was supported through the Duke Endowment Grant (7039-SP).

Educational intervention****

Educational content was developed and reviewed by a multidisciplinary radiation oncology team consisting of radiation oncologists, medical physicists, radiation therapists, nurses, dosimetrists, and research staff. Four treatment-specific educational pathways were created for deep inspiration breath-hold (DIBH) breast radiation therapy, prostate radiation therapy, stereotactic radiosurgery (SRS), and general external beam radiation therapy (EBRT). General was used for patients receiving EBRT for disease sites other than DIBH, prostate, or SRS, for which site-specific IPEPs had not been developed.

Educational materials reviewed key components of the radiation therapy process, including consultation, simulation, treatment planning, treatment delivery, and supportive resources. Materials were reviewed for clinical accuracy and patient-centered communication before implementation. Educational materials were available in both immersive VR and flattened 360-degree video formats to improve accessibility and accommodate patient preference or intolerance to headset-based immersive viewing modalities. The educational program averaged approximately 20 minutes of standard educational video content with an additional average of seven minutes of immersive or flattened educational content. 

Study population****

All adult patients receiving EBRT in the Department of Radiation Oncology during the six-month pilot period (May 2025 through October 2025) were eligible for enrollment unless they had a cognitive impairment or a benign diagnosis. Because the IPEP educational videos were only available in English during the study period, only English-speaking patients were included. Patients randomized to the IPEP arm completed cybersickness screening before immersive video viewing. Patients with a high probability of cybersickness were transitioned to the flattened 360-degree video format rather than excluded from the study. Patients were recruited following routine radiation oncology consultation appointments. Patients who enrolled were then randomized to either SOC (as defined in the study design above) or immersive educational intervention. 

Patients assigned to the intervention arm completed a cybersickness screening before immersive content viewing (Appendices) [15]. Based on patient focus group feedback, patients who preferred not to participate in immersive VR viewing, or who screened positive for potential cybersickness concerns, were instead provided flattened video-based educational materials. Patients randomized to the IPEP arm received the educational pathway corresponding to their disease site. Disease-specific pathways were available for breast, prostate, and SRS. Patients with all other EBRT treatment sites received the general IPEP pathway, which was developed for disease sites without a dedicated site-specific module.

**Figure 1:**
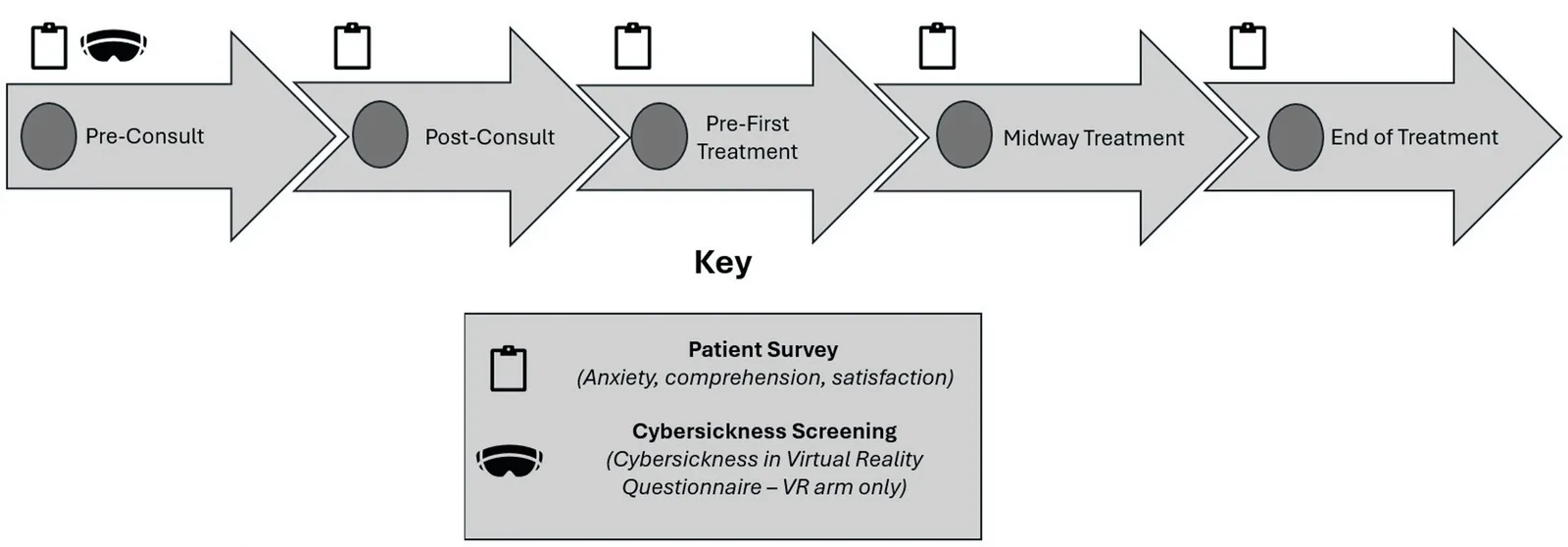
Timeline of survey administration Survey administration timeline illustrating administration of a multi-domain patient survey and physician-reported measures across key clinical milestones during radiation therapy VR: virtual reality

All educational materials were viewed in clinic to standardize exposure. The timeline shown in Figure 1 outlines the time point at which educational materials and survey materials were provided relative to the patient's care plan. 

Patient-reported outcomes****

Study-specific patient-reported survey instruments were developed by the research team based on concepts identified in prior literature related to patient education, treatment understanding, anxiety, and satisfaction. These instruments were not formally validated but were designed to capture preliminary patient-reported outcomes relevant to the feasibility evaluation of IPEP (Appendices). 

Patient surveys were collected at four predefined time points: pre-consult, post-consult, midpoint of treatment, and end of treatment. The pre-consult survey was administered immediately before the initial radiation oncology consultation during the patient's first visit to the department. The post-consult survey was administered following the physician consultation and delivery of any assigned educational intervention. The midpoint survey was administered during an on-treatment visit (OTV) occurring approximately halfway through the patient's prescribed radiation treatment course. The end-of-treatment survey was administered during the final OTV at completion of the radiation treatment course.

Clinical workflow metrics****

Clinical workflow metrics were extracted from the Varian ARIA oncology information system. Metrics included treatment duration, defined as the interval from Mode-Up Time to Plan Closure Time within ARIA, the number of setup images, the number of setup shifts, and composite translational and rotational shift magnitudes. These measures were evaluated longitudinally to assess trends in treatment efficiency and setup consistency. 

Statistical analysis****

Survey responses were summarized using descriptive statistics. Longitudinal changes were evaluated relative to pre-consult baseline values at predefined study time points. Between-group comparisons of change from baseline were performed using Wilcoxon rank-sum testing due to the ordinal nature of Likert-scale responses. Statistical significance was defined as p < 0.05. Workflow metrics were evaluated longitudinally using patient-level trend analyses to determine the proportion of patients demonstrating improvement over the treatment course. Given the pilot feasibility design, exploratory nature of the study, and limited sample size, statistical analyses were intended to identify preliminary trends and support hypothesis generation rather than provide definitive evaluation of clinical efficacy. Future studies with larger sample sizes will incorporate longitudinal statistical models that account for within-subject correlation across repeated measurements.

## Results

Participant characteristics****

**Figure 2:**
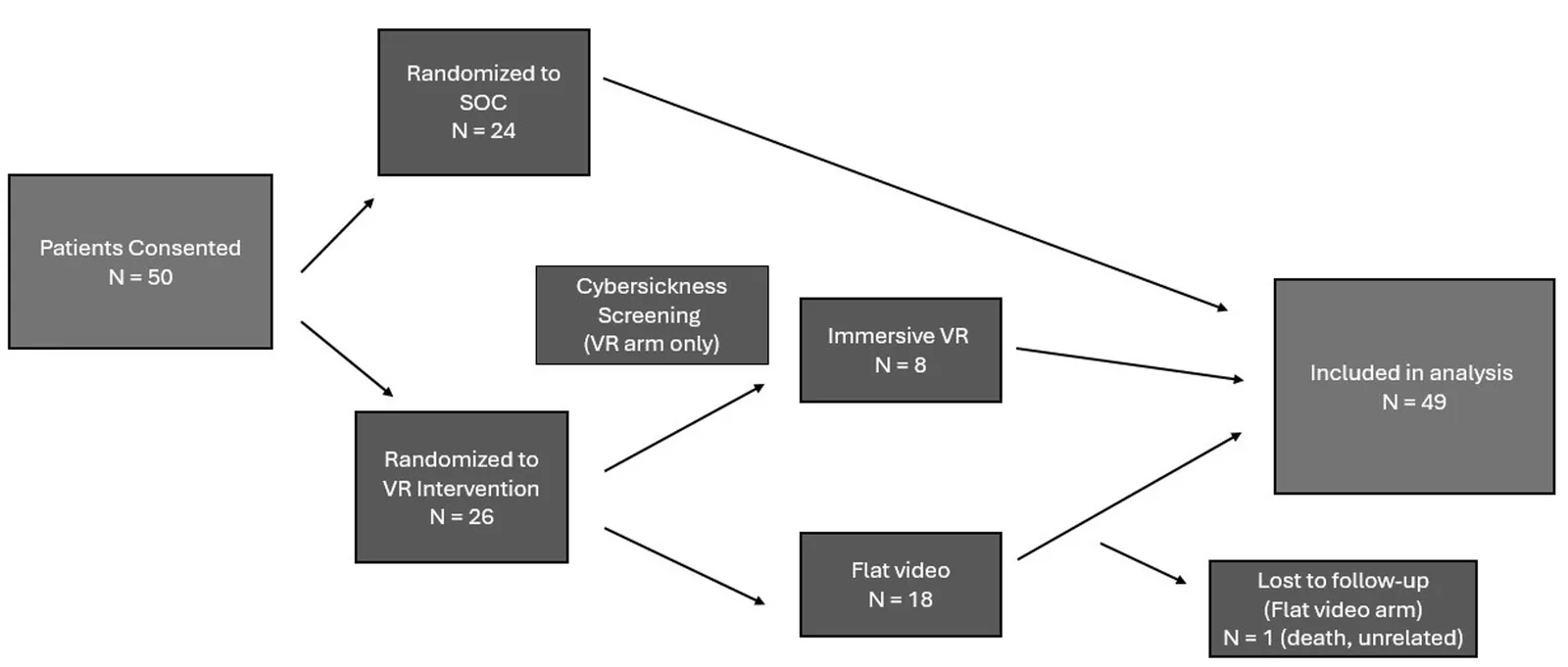
CONSORT flow diagram CONSORT: Consolidated Standards of Reporting Trials; SOC: standard of care; VR: virtual reality

Fifty patients consented to participate and were randomized to either SOC or immersive education. One patient was lost to follow-up for reasons unrelated to the intervention, resulting in 49 evaluable patients (24 SOC, 25 intervention). Baseline demographic characteristics were similar between groups. The mean patient age was 63 years, and the majority of patients underwent DIBH radiation therapy or general EBRT. Of the 25 intervention-arm participants, eight (32%) received headset-based immersive VR education, and 17 (68%) selected flattened 360-degree video delivery. No participants were assigned to flattened delivery because of cybersickness screening results; all participants receiving flattened video selected that modality based on preference. Patient demographics are summarized in Table 1, and the CONSORT (Consolidated Standards of Reporting Trials) diagram is presented in Figure 2.

**Table 1:** Patient demographics SOC: standard of care; VR: virtual reality; SD: standard deviation; DIBH: deep inspiration breath-hold; SRS: stereotactic radiosurgery

Characteristic	Overall (N = 49)	SOC (n = 24)	Enhanced education (n = 25; VR = 8, flat = 17)
Age, years, mean ± SD	63 ± 10.8	65 ± 10.5	61 ± 9.5
Female, n (%)	33 (67.3)	14 (58.3)	19 (76)
Race, White, n (%)	41 (83.7)	21 (87.5)	20 (80)
Race, Black or African American, n (%)	8 (16.3)	3 (12.5)	5 (20)
Ethnicity, Hispanic, n (%)	1 (2)	0 (-)	1 (4)
Public insurance, n (%)	26 (53.1)	13 (54.2)	13 (52)
Private insurance, n (%)	23 (46.9)	11 (45.8)	12 (48)
DIBH, n (%)	21 (42.9)	9 (37.5)	12 (48)
Prostate, n (%)	5 (10.2)	3 (12.5)	2 (8)
General, n (%)	18 (36.7)	9 (37.5)	9 (36)
SRS, n (%)	5 (10.2)	3 (12.5)	2 (8)

The educational intervention was implemented within routine clinical workflows across multiple treatment pathways without requiring modifications to standard treatment delivery processes, supporting the feasibility of integrating IPEP into routine clinical practice.

Patient-reported outcomes

A summary of all patient-reported outcomes is presented in Table 2. Each area is further assessed in the following sections. 

**Table 2:** Summary of patient-reported outcomes P-values were calculated using the Wilcoxon rank-sum test. Bold text indicates statistical significance IQR: interquartile range; SOC: standard of care; W: Wilcoxon rank-sum test statistic

Outcome	Baseline median (IQR), SOC	Baseline median (IQR), intervention	End-of-treatment median (IQR), SOC	End-of-treatment median (IQR), intervention	Median Δ, SOC	Median Δ, intervention	P-value	W
Treatment-related anxiety	4 (0)	4 (2)	4 (2)	2 (1)	-0.5	-1.5	0.10	121.5
Cancer-related anxiety	4 (1)	4 (2)	4 (0.75)	3 (1.75)	0	-1	0.22	146
Care plan-related anxiety	3 (2)	3.5 (1.25)	3 (2)	3 (2)	0	-1	0.01	61.5
Understanding of radiation therapy	3 (1)	3 (1)	5 (5)	5 (5)	1	2	0.03	190.5
Satisfaction with educational materials	4 (1)	4 (1)	5 (1)	5 (1)	0.5	1	0.36	97.5

Patient-Reported Anxiety

The intervention group demonstrated greater reductions in treatment-related, cancer-related, and care plan-related anxiety throughout the treatment course compared with SOC. Treatment-related anxiety decreased in both groups following consultation, with larger early reductions observed in the intervention arm. Although anxiety increased before treatment in both groups, lower treatment-related anxiety scores were observed in the intervention group at mid-treatment and end-of-treatment assessments. The median reduction in treatment-related anxiety from pre-consult to the end of treatment was greater in the intervention group compared with SOC (−1.5 vs. −0.5), though this difference was not statistically significant (W = 121.5, p = 0.10).

**Figure 3:**
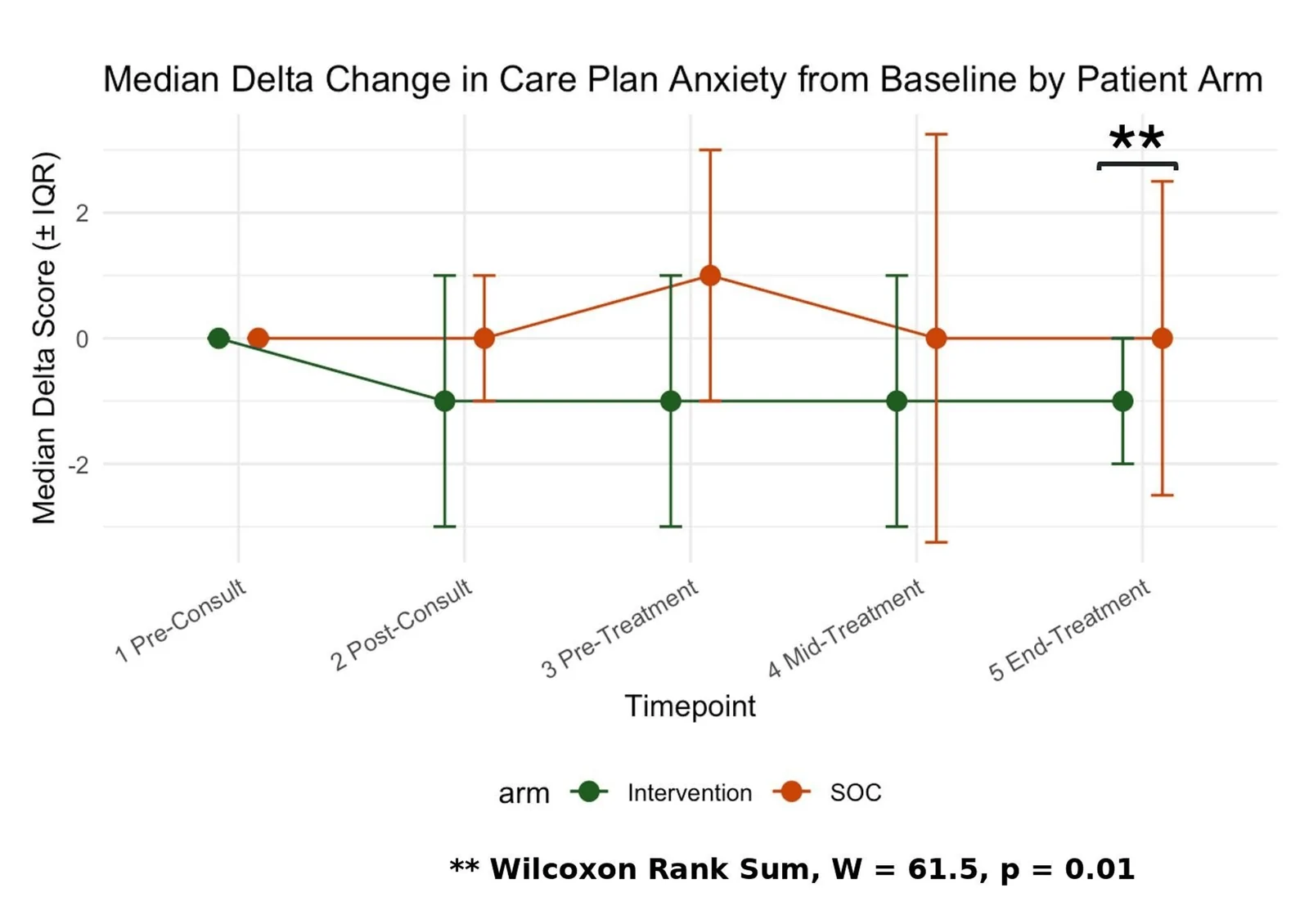
Care plan-related anxiety: change in median delta for care plan anxiety for each arm over time IQR: interquartile range

Cancer-related anxiety also decreased after consultation in both groups. At the end-of-treatment assessment, the intervention arm had lower mean and median cancer-related anxiety scores than the SOC arm. Median change from pre-consult to end of treatment favored the intervention arm (∆−1 vs. ∆0), though this difference was not statistically significant (W = 146, p = 0.22). The largest between-group difference was observed for care plan-related anxiety. The intervention arm began with higher baseline care plan-related anxiety than the SOC arm but demonstrated a larger reduction over time. Median reduction from pre-consult to end of treatment was significantly greater in the intervention arm than in the SOC arm (∆−1 vs. ∆0; W = 61.5, p = 0.01), as shown in Figure 3.

*Patient Understanding*****

**Figure 4:**
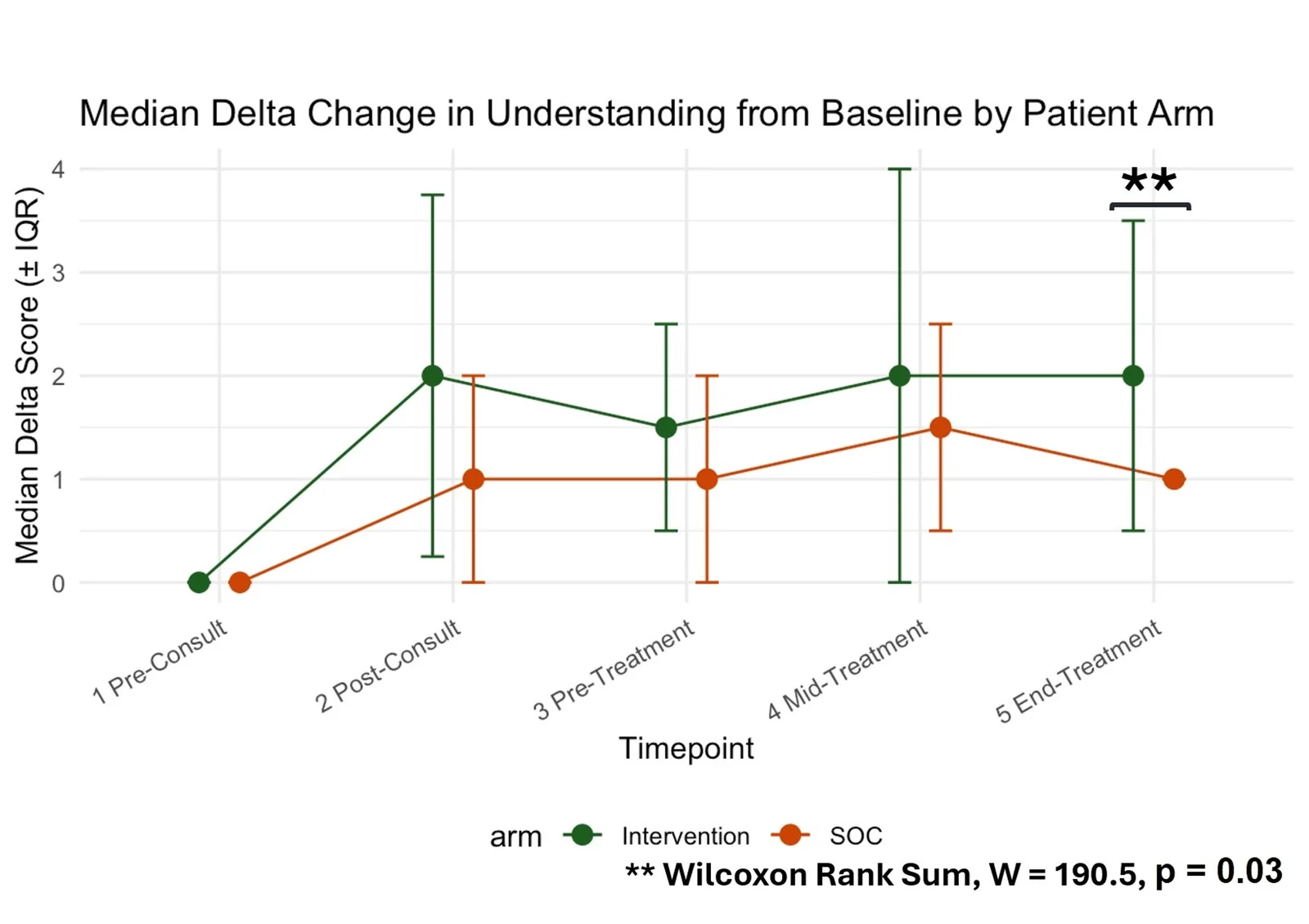
Patient understanding: change in median delta for understanding for each arm over time IQR: interquartile range

Patient-reported understanding of how radiation would be used to treat their cancer or condition increased in both groups following consultation. The intervention arm demonstrated a larger median improvement from pre-consult to end of treatment compared with the SOC arm (∆+2 vs. ∆+1), and this difference was statistically significant (W = 190.5, p = 0.03), as shown in Figure 4.

*Patient Satisfaction*****

Patient satisfaction improved in both groups throughout treatment. Both arms demonstrated increased satisfaction following consultation and generally favorable satisfaction scores throughout treatment. Median change in satisfaction from pre-consult to end of treatment was greater in the intervention arm than in the SOC arm (∆+1 vs. ∆+0.5), but this difference was not statistically significant (W= 97.5, p = 0.36). Additional modality-specific questions administered to intervention-arm participants demonstrated favorable perceptions of both immersive and flattened video-based educational materials. All responding intervention participants rated their educational modality favorably. A substantial proportion of intervention patients selected flattened video viewing rather than full immersive VR, supporting the importance of flexible educational delivery options during clinical implementation. 

*Qualitative Feedback*****

Limited qualitative feedback was collected. Responses from both study arms reflected positive perceptions of the educational experience. Participants receiving immersive education described the intervention as helpful and informative. 

Clinical workflow metrics****

Clinical workflow metrics were evaluated descriptively as exploratory outcomes, and the results are presented in Table 3.

**Table 3:** Summary of clinical workflow metrics SOC: standard of care; N/A: not evaluated

Metric	% Negative slope, SOC	% Negative slope, intervention	≥ 20% Reduction, SOC	≥ 20% Reduction, intervention
Treatment duration	68.2%	91.7%	75.0%	76.0%
Number of setup images	72.7%	79.2%	75.0%	80.0%
Number of setup shifts	40.9%	62.5%	54.2%	60.0%
Translational shift magnitude	59.1%	41.7%	N/A	N/A
Rotational shift magnitude	40.9%	54.2%	N/A	N/A

A greater proportion of intervention-arm patients demonstrated decreasing treatment duration over time compared with SOC patients, based on descriptive patient-level slope analysis (91.7% vs. 68.2%). No statistical comparison was performed for these exploratory workflow metrics. Similar directional trends favored the intervention arm for number of images and number of setup shifts. The proportion of patients achieving at least a 20% reduction in treatment duration was similar between groups. Results for composite translational and rotational shift magnitude were mixed, with the SOC arm showing a greater proportion of patients with decreasing translational shift magnitude and the intervention arm showing a greater proportion with decreasing rotational shift magnitude. Overall, workflow metrics suggested possible treatment-efficiency trends favoring the intervention arm for selected measures; however, these findings should be interpreted as exploratory and hypothesis-generating.

## Discussion

This prospective pilot feasibility study suggests that implementation of an IPEP may improve patient-reported outcomes during radiation therapy. For this study, our IPEP included either headset-based immersive VR or flattened 360-degree first-person video. Patients receiving immersive educational intervention demonstrated greater reductions in anxiety and greater improvements in treatment understanding compared with SOC education. The most pronounced improvement was observed in care plan-related anxiety, where the intervention group demonstrated significantly greater reductions over the treatment course. These findings suggest that immersive educational interventions may improve patient preparedness and familiarity with radiation oncology workflows. Improved patient understanding observed in the intervention group may represent a potential mechanism underlying reductions in anxiety. Prior studies have demonstrated associations between improved treatment understanding and reduced anxiety among oncology patients [3-5,7,16-19]. Immersive educational approaches may enhance understanding by improving visualization of treatment environments and procedural expectations. 

In addition to patient-reported outcomes, workflow metrics demonstrated exploratory trends toward improved treatment efficiency in the intervention arm. Greater proportions of intervention patients demonstrated reductions in treatment duration, imaging frequency, and setup shifts over time based on descriptive patient-level trend analyses. Although these findings were not statistically evaluated and should be interpreted cautiously, they suggest potential associations between immersive educational interventions and treatment workflow measures that warrant further investigation in larger, adequately powered studies. Our findings are consistent with prior studies evaluating immersive educational interventions in radiation oncology. Previous investigations have demonstrated improvements in patient understanding, engagement, and satisfaction following VR- and video-based educational interventions [3,5,10-13,18-19]. Unlike many prior studies, the present study prospectively evaluated an immersive educational intervention integrated into routine clinical workflow using a randomized design.

This study has several limitations. First, this was a single-institution pilot study with a limited sample size, which may reduce generalizability. The relatively small sample size and absence of an a priori power calculation limit the statistical power and generalizability of the findings. As this was a prospective pilot feasibility study conducted over a predefined six-month enrollment period, the primary objective was to evaluate the feasibility and preliminary impact of IPEP. Future studies with larger, adequately powered cohorts are needed to confirm these findings. Additionally, the use of simple randomization without formal allocation concealment may have led to group imbalances, particularly given the relatively small sample size. Future studies should consider more robust randomization approaches, such as block randomization or stratification based on relevant demographic and clinical characteristics, to minimize potential group imbalances and strengthen comparisons between study arms. Second, treatment pathways were not evenly distributed, with a large proportion of patients undergoing DIBH breast radiation therapy. Additionally, baseline care plan-related anxiety differed between study arms, which may have influenced the magnitude of observed longitudinal changes.

Third, the sample size did not permit direct comparison between immersive VR and flattened video participants within the intervention arm, limiting assessment of modality-specific effects. The proportion of patients selecting flattened video viewing also suggests that patient comfort and familiarity with immersive technologies may substantially influence implementation and scalability. Additionally, provider turnover during this study period may have introduced variability in patient experience, potentially influencing patient-reported outcomes. Similarly, this study included patients enrolled by six physicians and physician assistants, each with distinct consultation and education styles; therefore, education delivered within the SOC arm was not fully standardized. Fourth, patient-reported outcomes were assessed using study-specific Likert-scale survey items rather than previously validated domain-specific instruments. While these measures provided preliminary insights into patient perceptions of understanding, anxiety, and satisfaction, future studies should incorporate validated instruments to improve measurement consistency and comparability. Fifth, as with most patient education interventions, participants may have accessed additional educational resources outside of the study intervention. The amount and type of outside information reviewed before or during treatment were not controlled or quantified and may have influenced patient-reported outcomes. Finally, workflow metrics evaluated in this study represent surrogate indicators of treatment efficiency rather than direct measures of treatment compliance. 

Despite these limitations, this study demonstrates the feasibility of implementing immersive educational interventions within routine radiation oncology workflows. Feasibility was assessed descriptively through successful patient enrollment and completion of the intervention, integration of the intervention into standard clinical workflows, development and deployment of treatment-specific educational content, accommodation of multiple educational delivery modalities, and expansion of the program to additional clinical sites within the organization. 

Future studies should evaluate these interventions in larger and more heterogeneous patient populations while further assessing the relative impact of immersive versus standard video educational delivery. Additionally, formal evaluation of educational content quality using validated assessment tools, such as the Patient Education Materials Assessment Tool (PEMAT), to further characterize the understandability and actionability of IPEP educational materials should be performed. Implementation of IPEPs requires consideration of cost and scalability. The hardware used for this study, including VR headsets and supporting tablet devices, cost approximately $2,000 per educational station, excluding filming equipment and personnel time required for content development and workflow integration. 

To improve scalability and accessibility, the intervention was intentionally designed to support both immersive VR and flattened video-based delivery modalities. A substantial proportion of patients selected flattened video viewing rather than full immersive VR, suggesting that flexible delivery approaches may improve implementation feasibility while reducing barriers related to patient comfort, familiarity with technology, and potential cybersickness concerns. The educational materials developed during this study have since been expanded for use across additional clinical sites within the organization. Future implementation efforts will focus on broader electronic distribution through patient portal platforms to improve accessibility for patients and caregivers outside the clinic setting.

## Conclusions

A flexible IPEP was successfully integrated into routine radiation oncology workflows and was associated with improved patient-reported understanding and reduced care plan-related anxiety in this pilot randomized study. These findings support the potential value of patient-centered immersive educational approaches integrated into radiation oncology practice. The relatively low-cost and adaptable nature of this intervention may facilitate future implementation across a variety of radiation oncology settings. Larger multi-institutional studies are warranted to further evaluate the effectiveness of immersive educational programs, their impact on patient experience, and their potential influence on clinical workflow outcomes.

## Appendices

Appendix A - Cybersickness in Virtual Reality Questionnaire (CSQ-VR) 

The Cybersickness survey was adapted to score the probability that patients would be affected by cybersickness to offer an alternative viewing method. 

Cybersickness in Virtual Reality Questionnaire

Do you have concerns with viewing videos in virtual reality? If so, you can choose to view virtual reality videos on a flat screen instead.

Yes

No

Have you ever been diagnosed with epilepsy or are you predisposed to seizures?

Yes

No

If you answered yes to any of the above questions - END HERE

If you answered no to both questions - CONTINUE SURVEY BY ANSWERING QUESTIONS BELOW 

_____________________________________________________________________________ 

Please circle the response, from 1 to 7, that best corresponds to the presence and intensity of the symptom (Table 4).

**Table 4:** Symptom intensity

1	2	3	4	5	6	7
Absent feeling	Very mild feeling	Mild feeling	Moderate feeling	Intense feeling	Very intense feeling	Extreme feeling

Nausea A: Do you get nausea (e.g., stomach pain, acid reflux, or tension to throw)?

Please write below any additional comments relevant to the question above.

Nausea B: Do you get dizzy (e.g., feeling light-headed or spinning feeling)?

Please write below any additional comments relevant to the question above.

Vestibular A: Do you get disoriented (e.g., spatial confusion or vertigo)?

Please write below any additional comments relevant to the question above.

Vestibular B: Do you have balance troubles or feel imbalanced?

Please write below any additional comments relevant to the question above.

Oculomotor A: Do you have visual fatigue (e.g., eyes feel tired or sleepy)

Please write below any additional comments relevant to the question above.

Oculomotor B: Do you have visual discomfort (e.g., eyestrain, blurred vision, or headache)?

Please write below any additional comments relevant to the question above.

END OF PATIENT COMPLETION SECTION

FOR PROVIDER TO COMPLETE

**Table 5:** CSQ-VR scoring CSQ-VR: Cybersickness in Virtual Reality Questionnaire

Category	Symptom	Symptom intensity	Category score
Nausea	Nausea (nausea A)		
	Dizziness (nausea B)		
Vestibular	Disorientation (vestibular A)		
	Imbalance (vestibular B)		
Oculomotor	Fatigue (oculomotor A)		
	Discomfort (oculomotor B)		
CSQ-VR Score = total of each category			

Patient will be enrolled in arm IPEP - VR if: 

Patient answered NO to the concerns and epilepsy questions 

AND None of the category scores are greater than 10 

AND the total score is less than 25

If any of the above are not true, the patient will be enrolled in arm IPEP - Flattened.

Appendix B - survey provided to patients 

Use the following scale for all questions.

**Table 6:** Patient survey scale

1	2	3	4	5	NA
Strongly disagree	Disagree	Neutral	Agree	Strongly agree	Not applicable

I know how radiation will be used to treat my cancer or condition.

I am pleased with the teaching materials given to me about my care.

I am nervous about my cancer or condition.

I am nervous about my treatments.

I am nervous about the plan for my care visits.

The teaching videos were helpful.

I found the virtual reality videos to be helpful.

The virtual reality videos made me uneasy, have headaches, or nausea.

Please write below any additional comments relevant to the questions above:
